# An Atypical Calcaneal Fracture in a Child: A Literature Review Concerning the Treatment

**DOI:** 10.14740/jocmr1977w

**Published:** 2014-10-16

**Authors:** Leonardo Waihrich Guterres, Deryck Aguiar Ribeiro, Tiango Aguiar Ribeiro

**Affiliations:** a Servico de Ortopedia e Traumatologia do Hospital Universitario de Santa Maria (SOT-HUSM), Universidade Federal de Santa Maria (UFSM), Santa Maria, Rio Grande do Sul (RS), Brazil; b Estudante de Medicina da Universidade de Santa Cruz do Sul (UNISC), Santa Cruz, Rio Grande do Sul (RS), Brazil; c Programa de Pos-Graduacao em Ciencias da Saude, Centro de Ciencias da Saude (CCS), Universidade Federal de Santa Maria (UFSM), Santa Maria, Rio Grande do Sul (RS), Brazil

**Keywords:** Calcaneal fracture, Child, Computed tomography

## Abstract

Calcaneal fractures are considered uncommon accounting for 0.005-0.41% of all children fractures. Few reports concerning treatment are available. Most of these fractures are non-displaced/minimally displaced and are associated with a fall of less than 1 m. The aim of this case report was to discuss the diagnosis and treatment of a child calcaneal fracture, an atypical presentation despite the high energy mechanism of trauma. A 7-year-old child fell from a 5-m ladder with all his weight on his right heel. Significantly hind-foot reduced range of motion associated with a lateral/plantar calcaneal swelling and pain was found. Neurovascular examination and other parts of the body were normal. Radiograph showed an undisplaced calcaneal body fracture and computed tomography confirmed no subtalar joint involvement. A splint followed by plaster was applied. Weight bearing and deambulation were not allowed. After 4 weeks, no pain and limping was reported by the child’s parents. Plaster was removed and radiograph showed fracture consolidation. Patient had no complaints of pain, no restrictions in range of motion and normal walking. Limping in children is a difficult complaint to assess. Differential diagnoses of a calcaneal fractures should be performed, even without a history of trauma or a history of trivial trauma.

## Introduction

Tarsal fractures in children are considered extremely rare and account for less than 1% of all fractures in the childhood [1]. Calcaneal fractures are uncommon tarsal fractures accounting for about 0.005-0.41% [2-4]. Few reports in the literature concerning the fracture treatment are available [5-9]; the first case of this fracture in a child was reported in 1969 by Thomas [10], followed by another case by Moyson et al [11] in 1971. These publications have increased over the last decades [2, 12-17].

Most of calcaneal fractures are non-displaced [7] or minimally displaced [17] due to the low body weight and higher amount of cartilage [13] associated with the most common mechanism of trauma: fall of less than 1 m [9]. Seventy-five percent of calcaneal fractures are extra-articular [18] and intra-articular fractures are considered even more rare [17]. The aim of this case report was to relate and discuss the diagnosis and the treatment of a calcaneal fracture in a child which is an atypical presentation despite the high energy mechanism of trauma.

## Case Report

A 7-year-old child previously healthy has fallen from a ladder of 5 m on which the child has risen to play in a small time of carelessness of parents. He was found by his parents crying and limping and reported pain in the right hind-foot region. Immediately parents sought our medical emergence service. Following a long anamnesis it was concluded that patient has fallen standing with all his weight on his right heel. The child did not report pain in any other part of his body.

A prolonged and detailed physical examination was performed. Lateral and plantar calcaneal swelling were found. No hematoma was present. Limping and pain was also present. During examination, the patient had significantly hind-foot reduced range of motion associated with pain. No abrasion and skin lacerations were present. Neurovascular examination demonstrated no abnormalities.

**Figure 1:**
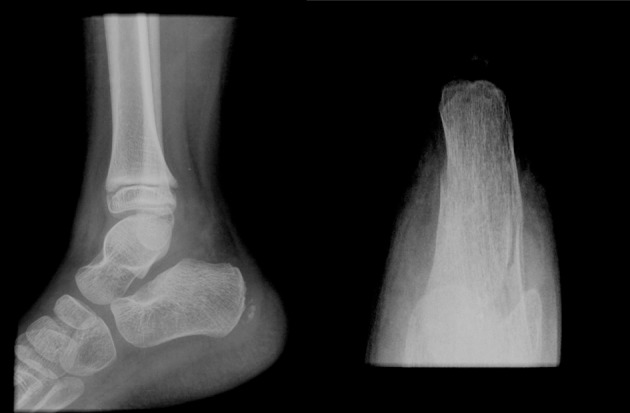
Lateral and axial image of the right calcaneal. Lateral view has not shown signs of fracture. The axial view revealed a calcaneal body fracture. Both images do not clarify if the fracture affected the joint.

**Figure 2:**
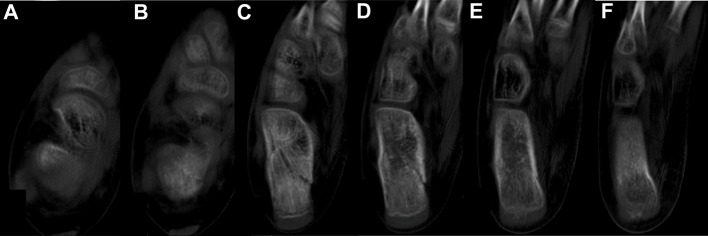
Computed tomography images demonstrate: (A, B) axial slices showing the end of the subtalar joint; (C-F) sequential axial slices from proximal to distal showing the fracture of the calcaneal body and not showing articular fracture.

Radiographic examination (Fig. 1) showed in an axial view an undisplaced calcaneal body fracture. An ankle splint was applied with the foot maintained in a neutral position (90° of flexion). In order to exclude the involvement of the subtalar joint, an elective computed tomography (CT) was solicited. One week after injury, the parents followed the child for review. No complaint was referred by the patient and the CT was evaluated (Fig. 2). No subtalar joint involvement was found and an extra-articular calcaneal body fracture was confirmed. The splint was replaced by a plaster. Weight bearing and deambulation were not allowed to the patient.

**Figure 3:**
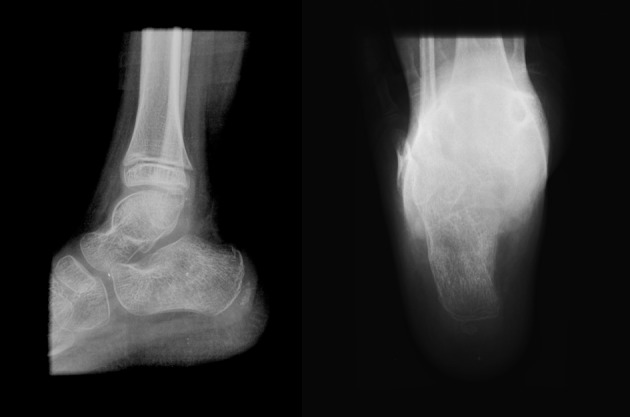
Lateral and axial image of right calcaneal 4 weeks post-treatment demonstrating the fracture consolidation.

After 4 weeks of immobilization, the child returned with his parents. Pain was not reported by the patient. Although deambulation has not been recommended, parents related that the child walked in the last 2 weeks. They also reported that they did not observe pain and limping. The plaster was removed and a radiograph was performed. The images demonstrated calcaneal fracture consolidation (Fig. 3).

The patient had no complaints of pain, no restrictions in ankle range of motion and a normal walking.

## Discussion

Limping in children is a difficult complaint to assess [19]. It is considered a challenge to the orthopedic surgeons [20, 21], and surgeons should know the different pathologies that cause limping [19]. In all limping children, calcaneal fractures should be suspected even without trauma history [5]. The delay in foot fracture diagnosis in children is not unusual [3] and may be estimated in 27-55% [8, 9, 22]. Therefore the incidence of these fractures may be higher than expected [7, 9, 23]. For fracture diagnosis, the simple X-ray is considered the choice. However in limping children with hind-foot pain with a normal X-ray, the additional image exams should be taken. Scintigraphy was used in the recent past [5, 24], but currently the choices are CT [3] or magnetic resonance [14], with CT being the best method [18]. In our opinion and as proposed by Price et al [18], the additional image exams were also important to diagnose non-displaced articular fractures not viewed in the normal X-ray.

Fractures of the calcaneous in children younger than 10 years in the vast majority of cases are the result of low-energy injuries [17] and may be considered as falls of less than 1 m [9]. Though in some cases the history of trauma may not be documented [5]. Because of this mechanism of trauma, we believe that fractures have specific patterns, such as extra-articular non-deviated or minimally deviated fractures. In the older children, the most common mechanism of trauma is falls from greater heights, being reported an average height of 4 m [9]. In these cases, the fracture characteristics are extra-articular deviated fractures or intra-articular fractures, similar to those found in adults [17].

Associated injuries are present in 57% of all cases. Commotio cerebri syndrome and fractures of the talus, spine and pelvis were reported [3, 6, 22]. We believe that these injuries are very closely related to a high energy mechanism of trauma. In this case report despite the high energy trauma none associated injuries or fractures were diagnosed.

The treatment to calcaneal fractures in children is usually conservative [15] and should be employed in most of the cases, even for severe fractures [3]. Undoubtedly for all non-displaced or minimally displaced extra-articular calcaneal fractures (minimally displaced fractures can be considered those with 1 - 2 mm of deviation between the fragments [12]), the conservative treatment is the choice [12, 13, 16, 23]. Several authors have reported good outcomes in these cases [9, 12, 15, 23]. A cast immobilization for 4 - 6 weeks was the choice as performed in this case and progressive weight bearing should be employed [18].

To articular or extra-articular deviated calcaneal fractures, the non-operative treatment may be an option as advocated by Thomas [10], who believes that children under the age of 10 years have an excellent remodeling potential. In these cases, the immature talus grows on the deformed surface of the calcaneous and fits, forming a congruent subtalar joint. However some authors reported unsatisfactory results with conservative treatment [8, 22, 25] and thus the surgical treatment became the choice [17, 26-28] resulting in good postoperative outcomes [27, 29]. The surgical treatment options described are: 1) open reduction and internal fixation with screws and plate (small and mini-fragments) [16, 17]; 2) open reduction and fixation with Kirschner wires [3] or screws [2]; and 3) minimally invasive approach and fixation with K-wires or screws [2]. But surgical approach can have its disadvantages. Injury to the periosteum may lead to a malunion and delay in healing, as well as infection and chronic postoperative pain [15].

In conclusion, in this case, even though the mechanism of injury was a high energy trauma, the fracture pattern was extra-articular which is quite unusual and conservative treatment was successful employee. An important message remains: in all limping children calcaneal fracture may be a diagnosis and deserves investigation, even without a history of trauma or a history of trivial trauma.
